# Conceptualising the initiation of researcher and research user partnerships: a meta-narrative review

**DOI:** 10.1186/s12961-020-0536-9

**Published:** 2020-02-18

**Authors:** Maria Maddalena Zych, Whitney B. Berta, Anna R. Gagliardi

**Affiliations:** 1 Institute of Health Policy, Management and Evaluation, University of Toronto, 155 College Street, Suite 425, Toronto, Ontario M5T 3M6 Canada; 2 Toronto General Hospital Research Institute, University Health Network, 200 Elizabeth Street, 13EN-228, Toronto, Ontario M5G 2C4 Canada

**Keywords:** Integrated knowledge translation, research collaboration, partnership initiation, meta-narrative review

## Abstract

**Background:**

Integrated knowledge translation refers to researcher and research user partnerships to co-generate and implement knowledge. This type of partnership may be critical to success in increasing knowledge use and impact, but the conceptualisation of its initiation has not been fully developed. Initiating this type of partnership has proven to be challenging but crucial to its success. The purpose of this study was to conduct a meta-narrative review of partnership initiation concepts, processes, enablers, barriers and outcomes in the disciplines of healthcare and social sciences where examples of researcher and research user partnerships were found.

**Methods:**

Seven research traditions were identified. Three were in the discipline of social sciences (including psychology, education and business) and five were in the discipline of healthcare (including medicine, nursing, public health, health services research). Searches were conducted in MEDLINE, EMBASE, CINAHL, ABI Inform, ERIC, PsychInfo and the Cochrane Library on June 9, 2017. Fifty titles and abstracts were screened in triplicate; data were extracted from three records in duplicate. Narratives comprised of study characteristics and conceptual and empirical findings across traditions were tabulated, summarised and compared.

**Results:**

A total of 7779 unique results were identified and 17 reviews published from 1998 to 2017 were eligible. All reviews identified a partnership initiation phase referred to as ‘early’ or ‘developmental’, or more vaguely as ‘fuzzy’, across six traditions – integrated knowledge translation, action research, stakeholder engagement, knowledge transfer, team initiation and shared mental models. The partnership initiation processes, enablers, barriers and outcomes were common to multiple narratives and summarised in a Partnership Initiation Conceptual Framework. Our review revealed limited use or generation of theory in most included reviews, and little empirical evidence testing the links between partnership initiation processes, enablers or barriers, and outcomes for the purpose of describing successful researcher and research user partnership initiation.

**Conclusions:**

Narratives across multiple research traditions revealed similar integrated knowledge translation initiation processes, enablers, barriers and outcomes, which were captured in a conceptual framework that can be employed by researchers and research users to study and launch partnerships. While partnership initiation was recognised, it remains vaguely conceptualised despite lengthy research in several fields of study. Ongoing research of partnership initiation is needed to identify or generate relevant theory, and to empirically establish outcomes and the determinants of those outcomes.

## Background

Partnerships between researchers and research users, who may be policy-makers, managers, clinicians or patients, to co-generate and implement knowledge have become prevalent in implementation research and practice [1, 2]. Such partnerships empower research users, who traditionally were the subjects of research or passive consumers of its outcomes, to shape research such that its conduct and outputs are more relevant and likely to be used [3]. Consequently, research based on regional and national initiatives has demonstrated beneficial outcomes arising from researcher and research user partnerships. For example, family caregivers and caregiver associations in Canada met with researchers, clinicians, healthcare administrators and policy-makers, and identified caregiver needs to inform a research agenda [4]. In a region of Quebec, Canada, a collaboration between researchers and representatives of family medicine groups resulted in implementation of a chronic disease prevention and management programme that engaged nurses, nutritionists, pharmacists, psychologists and medical specialists, and improved various outcomes (self-management, fruit and vegetable consumption, physical activity and quality of life) among patients at risk for cardiovascular disease, chronic obstructive pulmonary disease, asthma and diabetes [5]. In some jurisdictions, researcher and research user partnerships have become routinised in the structures and processes relied upon to generate and implement research. The Netherlands invested over 30 million euros to establish 11 Academic Collaborative Centres between 2006 and 2014, which involved partnerships between healthcare organisations and universities, leading to 150 short- and long-term policy-driven projects to improve public health [6]. Similarly, in England, 200 million pounds were invested to create 9 Collaborations for Leadership in Applied Health Research and Care (CLARHCs) between 2008 and 2013, which are regional partnerships between healthcare organisations and universities that improved healthcare delivery (i.e. recording and management of blood pressure, screening and prevention of venous thromboembolism) and patient outcomes (i.e. increased confidence in home-based self-management for chronic obstructive pulmonary disease) [7].

Thus far, research has focused on identifying enablers and barriers of researcher and research user partnerships. A realist review of 23 studies of participatory research partnerships published up to 2011 identified numerous conditions and processes essential for partnership, including forming an advisory board, establishing programme and research protocols, developing capacity among team members to understand each other, and introducing mechanisms that foment discussion on conflicting ideas [8]. A scoping review of 13 studies of researcher and research user partnerships published from 2005 to 2014 identified these and additional enablers of partnership, including multiple and varied structured opportunities for interaction among partners, strong leadership and achieving early successes [9]. However, numerous challenges may preclude establishing collaborative relationships between partners or achieving research-informed policy or practice, resulting in token or asymmetric partnerships, meaning that not all partners were thoroughly engaged [10]. Identified barriers to partnership included differing needs and priorities between researchers and research users; lack of understanding about and skill for partnering among participants; failure to establish goals, roles and expectations; an absence of adequate funding or infrastructure to support partnership; and systemic disincentives for researchers and research users to engage in partnership [9]. This accumulated research suggests that researcher and research user partnerships experience barriers from the outset, during the research process, and through to dissemination and implementation of co-generated research but does not clearly distinguish barriers specific to different stages of partnership.

Extant research suggests that initiation of partnerships – early processes undertaken to establish and develop a partnership between researchers and research users – is a distinct stage in the continuum of a partnership that may be critical to its subsequent success [9, 11]. In our scoping review of the IKT literature, most included studies evaluated partnerships that were a minimum of 2 years from launch, yet they were beset by challenges reflecting partnership development [9]. Other research complemented this observation, showing that certain processes at an early stage of partnership formation were important to the further development of the partnership; for example, establishing convenient virtual and physical communication spaces and channels [9, 12, 13]; clarifying and establishing vision, mission, goals, terms of reference, rules, regulations, policies, priorities, strategic planning documents and project timelines [9, 13, 14]; negotiating roles, identifying member skills and delegating work [9, 14]; and ensuring clear leadership and engagement of team members [13–15]. Specific partnership initiation processes (i.e. creating an inventory of partner skills) may be associated with intermediate outcomes, such as ensuring research user engagement through the partnership process, that are, in turn, essential to long-term partnership outcomes such as the co-generation and use of research and improved clinical outcomes. In other words, the benefits of researcher and research user partnerships may not be realised if flourishing partnerships are not established at the outset.

While previous reviews [9] and concept analyses [10, 11, 16, 17] of researcher and research user partnerships have been published, none of those specifically assessed partnership initiation in depth. Bush et al. [17] concluded that co-creation partnerships, that is, partnerships to co-create knowledge, initiated by research user organisations appear to have greater beneficial outcomes than consultative type partnerships initiated by either universities or research user organisations. Their study focused on comparing types of partnerships with research users, namely consultative versus knowledge co-creation partnership [17], and they did not focus on the enablers and barriers specific to initiation, which is the scope of this paper. Given the demonstrated benefits of researcher and research user partnerships on research-informed policy and practice, further insight is needed to identify the conditions and processes that are essential to productive partnerships. Here, we report on a review of published research to describe how partnership initiation has been conceptualised, operationalised and evaluated in various fields of study. Our aim was to identify how to launch researcher and research user partnerships that are enduring and successful. In this review, we included articles from health services research and from other fields of study in the interest of generating a broader understanding of this phenomenon. Formally, the purpose of this study was to conduct a meta-narrative review of partnership initiation, including conceptualisation, processes, enablers, barriers and outcomes.

## Methods

### Approach

A meta-narrative review was conducted to describe partnership initiation across diverse literatures [11]. A meta-narrative review was first used by Greenhalgh et al. [18] in 2005 to explain disparate data encountered in their review of the diffusion of innovations in healthcare organisations. The goal of a meta-narrative review is sense-making; it seeks to identify, understand and describe relevant narratives, and synthesise and compare them in an over-arching narrative [19]. A meta-narrative is formally defined as a relatively new method of systematic review, designed for topics that have been conceptualised and studied in different ways by different groups of researchers [19]. The first part of a meta-narrative review involves seeking out different research traditions, or fields of study, and collect data that describes how a concept, in this case partnership initiation, was conceptualised [19]. In this study, the ‘narrative’ refers to how the idea of partnership initiation was conceptualised in each research tradition by noting terms used to describe initiation and the overall approach to partnership initiation, specifically the processes, barriers and enablers of initiation. The steps of a meta-narrative review include scoping the literature, searching for and selecting documents, and data extraction, analysis and synthesis [19]. A meta-narrative review is meant to be an iterative process, whereby steps such as searching or data extraction are modified prospectively as a deeper understanding of the topic is gained. We adhered to the following guiding principles of meta-narrative reviews: pragmatism (by exploring partnership initiation in a variety of traditions), pluralism (by considering studies of various designs included in reviews of different types), contestation (by comparing data from different traditions to generate higher-order insights), reflexivity (by documenting reflective insights and decisions) and peer review (by publishing and sharing the findings) [19]. We did not employ a theoretical orientation; instead, as is commonly done in meta-narrative reviews, we reported concepts, theories and descriptions as reported reviews in each research tradition. However, those details were compiled in narratives for each tradition, then compared across traditions and finally consolidated in a conceptual framework, which serves as early theory of IKT initiation. We used the Realist And Meta-narrative Evidence Syntheses: Evolving Standards (RAMESES) reporting standards [19] (Additional file 1).

### Scoping the literature

The scoping step involved browsing to identify different research traditions, or fields of study, and consulting with experts to support decision-making about which traditions to include. The literature was identified using an iterative process that yielded studies of partnership initiation. First, MZ searched the healthcare literature on partnerships to gain an understanding of the topic and begin conceptualising partnership initiation. This included sources known to the research team, including a review [9], books [2, 20], concept papers [1, 3, 12], evaluations of the CLARHCs [7, 15, 21, 22] and Academic Collaborative Centres [23], and a MEDLINE search for researcher and research user partnership literature. This step identified several labels used to refer to partnerships, for example, university–community partnerships, collaborative partnerships, teamwork and community-based participatory research. Next, MZ used the database Scopus to identify the traditions corresponding to articles that were most cited when searching for partnerships labelled using the aforementioned terms. These included the traditions of psychology, education and business from the disciplines of social sciences, and medicine, nursing, public health and health services research from the discipline of healthcare. MZ then conducted searches in MEDLINE, ABI/Inform, ERIC and PsychInfo using search terms including, but not limited to “translational medical research” or “community-based participatory research” and keyword counterparts for studies of partnership in medicine, nursing, education, psychology and organisational management literature. Reviewing search results revealed additional terms for researcher and research user partnerships: shared mental models, engaged scholarship, community-based action research, action research, stakeholder engagement, knowledge transfer or use, knowledge co-production, transdisciplinarity, working together, membership on a research team, partnership, or collaboration. The research team met in person and by teleconference on 15 occasions to establish which fields of study and corresponding databases were likely to yield research on partnership initiation, to further develop the search strategy, to generate initial eligibility criteria, and to discuss concepts, assess scoping review findings, and make a final selection of partnership-like concepts to be included in the review.

### Eligibility criteria

Inclusion criteria followed the PICO (Population, Issue, Comparisons, Outcomes) framework [24]. Eligibility criteria were informed by those employed in our previous scoping review [9], then refined concurrent with screening, which included an iterative process between identifying new concepts describing partnerships between researchers and research users, and editing our definitions of the PICO accordingly until we felt that our description was comprehensive. ‘Populations’ referred to researchers, labelled in studies as researchers, investigators or scientists, who conducted research of any design on any clinical, management or policy topic, and who work in any research setting such as a university or research institute, and/or research users, labelled in studies as clinicians, technicians, managers, policy-makers, decision-makers or others who used research, and who worked in any setting that governs, plans, oversees, monitors or delivers services or products. Researchers and research users could have similar or different professional positions, specialties or domains of knowledge/expertise. The ‘issue’ referred to partnership initiation, defined as establishing collaboration between researchers and research users with the intent of creating and implementing knowledge [9]. This included establishing collaboration goals, rules and processes through a variety of in-person or remote, synchronous or asynchronous communication [25, 26]. With respect to ‘comparisons’, studies described or evaluated one or more partnerships and reported on partnership initiation experiences, processes, enablers, barriers or outcomes; means of interaction between researchers and research users for initiation; the conditions or processes by which partnerships were initiated; or interventions designed to promote or support partnership initiation or any of its processes, for example, educating or training to help researchers and research users form strong partnerships. Enablers were defined as conditions, processes or other factors that facilitated or had a positive effect on partnership initiation. Barriers were defined as conditions, characteristics or other factors that challenged or had a negative effect on partnership initiation.

In our previous scoping review, we found that the literature on researcher and research user partnerships was not well-indexed, necessitating screening of more than 14,000 titles and abstracts [9]. Therefore, to enhance feasibility of this research, we restricted publication type to systematic reviews or meta-analyses of partnerships. More specifically, we included reviews according to a typology described by Pare et al. [27] as narrative, descriptive, scoping, qualitative, umbrella, theoretical, realist or critical reviews.

‘Outcomes’ included any reported consequence of partnership initiation, including but not limited to researcher or research user awareness, acceptance, attitude, knowledge, skill, competency, participation, satisfaction, behaviour, practice, processes, or team, organisational or system/population-level impact.

Reviews were not eligible if they lacked detail such that it was unclear if research users participated in research activities; involved for-profit academic–industry partnerships (because the commercial nature of these types of partnerships would necessitate that they be subjected to laws, regulations and explicit standardised processes related to industry); anecdotally described partnership planning or development but without empirical evaluation; concluded that research and research user partnerships were needed without having described and evaluated them; focused on issues of authorship among research collaborators; examined online communities where interaction or data were collected by social media; did not describe research methods; or did not provide or report details specific to partnership initiation.

### Searching

Several databases were searched for reviews of partnerships in healthcare and social sciences: MEDLINE (1946 to June 2017), EMBASE (1947 to June 2017), CINAHL (1937 to June 2017), ABI Inform Business Database (1971 to June 2017), ERIC (1966 to June 2017), PsychInfo (1806 to June 2017), and Cochrane Library (on June 9, 2017), which includes systematic reviews by the Joanna Briggs Institute and WHO, among others [28]. The search strategy was created by a librarian (MZ) according to the Peer-Review of Electronic Search Strategies guidelines [29] and adapted to each database’s thesaurus and/or indexing system. Searches were initially performed on August 11, 2016, and updated 11 times due to the iterative process of reading and discovering new synonyms referring to partnerships in the different traditions. Throughout the literature scoping process, the subject databases were narrowed down to those that included the most studies on researcher and research user partnerships, including healthcare, business, education, psychology, organisational management, knowledge management and information systems. The final searches were conducted on June 9, 2017, and the MEDLINE search is available in Additional file 2.

### Screening

Titles and abstracts were exported to EndNote X7 and duplicates were removed. To pilot test the screening process, MZ, WB and ARG independently screened a sample of 50 titles and abstracts from the MEDLINE results. No discrepancies were identified. MZ proceeded to screen remaining titles and abstracts and, when uncertain, reviewed decisions with ARG.

### Data extraction

A data extraction form was developed to collect the characteristics of each review (discipline, tradition, author, year of publication, country of first author), conceptual details commonly extracted in meta-narrative reviews that form the basis of each narrative (labels for partnership initiation, key actors, philosophical and/or research origins, conceptual or theoretical issues) [19], and details about partnership initiation that were empirically examined (processes, enablers, barriers, outcomes). To pilot test data extraction, MZ and ARG independently analysed 23 reviews at full-text level and extracted data from 3 included reviews. They compared and discussed findings. Discrepancies were minor and resulted in clarification of the level of detail of information MZ was to extract. MZ extracted data from the remaining reviews and tabulated the data. Data extraction was independently reviewed by ARG on three occasions. Methodological quality appraisal is not a requirement for a meta-narrative review [19]. However, to address this issue, we noted whether included reviews followed reporting criteria or checklists, which are meant to enhance the conduct and reporting of research (Table 1).

**Table 1:** Characteristics of included reviews

Study	Type of review	Time span of included studies	Discipline	Field of study	Review guidelines or recommendations followed
Tremblay et al., 2017, Canada [30]	Theoretical	Up to 2015	Social Sciences	Psychology	Framework synthesis
Gagliardi et al., 2016, Canada [9]	Scoping	2005–2014	healthcare	Health services research	Preferred Reporting Items for Systematic Reviews (PRISMA)
Salsberg et al., 2015, Canada [31]	Critical	1995–2009	Healthcare	Public health	None
Esmail et al., 2015, United States [32]	Critical	2005–2013	Healthcare	Health services research	None
Concannon et al., 2014, United States [33]	Systematic	2002–2013	Healthcare	Medicine	PRISMA
Filieri & Alguezaui, 2014, United Kingdom [34]	Systematic	1992–2012	Social sciences	Knowledge management	None
Andrews et al., 2012, United States [35]	Systematic	1995–2011	Healthcare	Nursing	Integrated review methodology
Jagosh et al., 2012, Canada [8]	Realist	1970–2011	Healthcare	Health services research	None
Orem et al., 2012, Uganda [36]	Descriptive	2000–2010	Healthcare	Public health	None
De-Pinho Campos et al., 2011, Canada [37]	Systematic	1990–2010	Healthcare	Public health	The Joanna Briggs Institute for Evidence Based Nursing and Midwifery’s An introduction to systematic reviews
Chiasson et al., 2009, United Kingdom [38]	Systematic	1982–2005	Social sciences	Information systems	None
Suarez-Balcazar et al., 2005, United States [39]	Theoretical	1977–2004	Social Sciences	Psychology	None
Guzman & Wilson, 2005, Australia [40]	Theoretical	1979–2003	social Sciences	Knowledge management	None
Riley-Tillman et al., 2005, United States [41]	Narrative	1977–2005	Social sciences	Education	None
Druskat & Pescosolido, 2002, United States [42]	Theoretical	1986–1996	Social sciences	Organisational management	None
Waterman et al., 2001, United Kingdom [43]	Systematic	1975–1998	Healthcare	Health services research	NHS’s Centre for Reviews and Dissemination Recommendations
Israel et al., 1998, United States [44]	Narrative	1968–1997	Healthcare	Public health	None

### Data analysis

Through repeated reading and analysis of quantitative and qualitative data extracted from each review, MZ prepared narratives that emerged across the traditions, which were labelled by the same language used to describe partnerships between researchers and research users in each research tradition. For example, the label ‘action research’ was used to organise narratives from all of the reviews that used this term to describe researcher and research user partnerships or a derivative term, such as community-based participatory research, community-based research, community organisations, etc. ARG and WB reviewed narratives on three occasions, and MZ refined the narratives with their feedback. MZ synthesised narratives conceptually using three techniques: paradigm bridging to identify common features, paradigm bracketing to identify differences, and meta-theorising to explore tensions or patterns across narratives in labels for partnership and partnership initiation, key actors, philosophical and/or research origins, and conceptual or theoretical issues. Content analysis of empirical findings extracted from each review was used to identify partnership initiation processes, enablers, barriers and outcomes. Conceptual and empirical findings associated with each narrative were tabulated, and summarised and compared in tabular and narrative format. Processes were described using action verbs to capture the fact that an activity or task was necessary. Enablers and barriers were described as nouns either positively or negatively effecting partnership initiation.

To generate an early theory of IKT initiation, we consolidated conceptual and empirical findings from narratives across traditions in a conceptual framework of how actors and processes may be influenced by enablers and barriers, and the potential associated outcomes. WB and ARG independently reviewed the findings and, through discussion with MZ, arrived at the final analysis.

## Results

### Search results

**Fig. 1:**
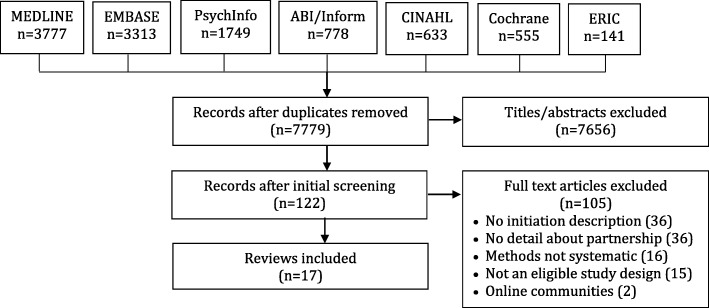
PRISMA flow chart describing screening of papers. This figure is a visual representation in flow chart that describes how many papers were excluded at each step of the meta-narrative review screening by the authors. It is based on Moher et al.’s [45] PRISMA flow chart for systematic reviews and meta-analysis

A total of 7779 unique records remained following the removal of duplicates in the search results from different databases. Screening of titles and abstracts excluded 7656 records. Screening of 122 full-text reviews excluded another 105 items for the following reasons: no description of initiation (*n* = 36), no or little conceptual or empirical detail about partnership (*n* = 36), review methods were not systematic (*n* = 16), item was not a review (*n* = 15) and the item pertained to online communities (*n* = 2). A total of 17 reviews were eligible for inclusion (Fig. 1). The data extracted from each review is available in Additional file 3 [8, 9, 30–44].

### Characteristics of included reviews

Review characteristics are summarised in Table 1. The 17 reviews were published between 1998 and 2017. Nine of the 17 reviews were published between 2012 to 2017 [8, 9, 30–36]. Reviews included primary studies published from 1968 to 2015. Reviews were conducted in the United States (*n* = 7) [32, 33, 35, 39, 41, 42, 44], Canada (*n* = 5) [8, 9, 30, 31, 37], the United Kingdom (*n* = 3) [34, 38, 43], Australia (*n* = 1) [40], and Uganda (*n* = 1) [36]. The types of reviews were classified using Paré et al.’s [27] definitions of systematic (*n* = 6) [33–35, 37, 38, 43], theoretical (*n* = 4) [30, 39, 40, 42], narrative (*n* = 2) [41, 44] and critical (*n* = 2) [31, 32] reviews, followed by one each of descriptive [36], realist [8] and scoping [9] reviews. Ten reviews emerged from the healthcare literature in the traditions of medicine [33], nursing [35], public health [31, 36, 37, 44] and health services research [8, 9, 32, 43]. Seven reviews emerged from the social sciences literature in the traditions of the study of psychology [30, 39], knowledge management [34, 40], information systems [38], education [41] and organisational management [42]. Six reviews followed reporting criteria such as the Preferred Reporting Items for Systematic Reviews [9, 33], framework synthesis recommendations [30], integrated review methodology [35], the Joanna Briggs Institute for Evidence Based Nursing and Midwifery’s systematic reviews recommendations [37] and the National Health System’s Centre for Reviews and Dissemination Recommendations [43] (Table 1).

### Integrated knowledge translation (IKT) concepts described in narratives

Distinct traditions included IKT, action research, stakeholder engagement, knowledge transfer, team initiation and shared mental models. Conceptual issues including labels for partnership and partnership initiation, actors, the origin (field of study, philosophy, research), and concepts or theories underlying partnership corresponding to narratives are summarised and compared in Table 2.

**Table 2:** Conceptual details about partnership initiation

Study	Narrative	Labels		Key Actors		Origins (discipline/field of study)	Concepts or theories
		Partnership	Partnership initiation	Researchers	Research users		
Tremblay et al., 2017, Canada [30]	Action research	Community-based participatory research	First stage	Researchers	Community organisation, coalition of organisations	Social Sciences/Psychology	Social movement theory
Gagliardi et al., 2016, Canada [9]	Integrated knowledge translation	Integrated knowledge translation	Formation stage	Researchers	Organisation or system-level decision-makers, including clinician managers, health facility managers and policy-makers	Healthcare/health services research	NR
Salsberg et al., 2015, Canada [31]	Action research	Participatory research	Fostering a partnership	Researchers	Stakeholders, community members, end-users	Healthcare/Public Health	NR
Esmail et al., 2015, United States [32]	Stakeholder engagement	Stakeholder engagement research	Early stage	Researchers	Patients, public	Healthcare/health services research	NR
Concannon et al., 2014, United States [33]	Stakeholder engagement	Stakeholder engagement	Early stage	Researchers	Individual or group who is responsible for or affected by health and healthcare-related decisions	Healthcare/medicine	NR
Filieri & Alguezaui, 2014, United Kingdom [34]	Knowledge transfer	Structural social capital	Development stage, fuzzy front end	Researchers	Business managers, business partners, customers, suppliers, universities, competing firms	Social sciences/knowledge management	Social capital theory
Andrews et al., 2012, United States [35]	Action research	Community-based participatory research	Partnership development	Academic partners	Community partners	Healthcare/Nursing	NR
Jagosh et al., 2012, Canada [8]	Action research	Participatory research	Early stage	Researchers	People affected by issues under study and/or decision-makers who apply research funding	Healthcare/Health Services Research	NR
Orem et al., 2012, Uganda [36]	Integrated Knowledge Translation	Knowledge translation partnership	Pre-research stage	Researchers	Policy-makers	Healthcare/Public Health	NR
De-Pinho Campos et al., 2011, Canada [37]	Team initiation	Public–private partnerships	Development stage	Researchers	Government, hospitals, pharmaceutical and biotechnology companies, non-governmental organisations, foundations, experts, investors	Healthcare/Public Health	NR
Chiasson et al., 2009, United Kingdom [38]	Action research	Action research	Outset of activities	Researchers	Stakeholders, decision-makers	Social sciences/information systems	NR
Suarez-Balcazar et al., 2005, United States [39]	Action research	Community–university collaborations	Gaining entry into the community	Researchers, academics	Community members	Social sciences/psychology	NR
Guzman & Wilson, 2005, Australia [40]	Knowledge transfer	Inter- and intra-organisational knowledge transfer	‘Soft issues’ before developmental stage	Organisation or team members	Organisation or team members	Social sciences/knowledge management	NR
Riley-Tillman et al., 2005, United States [41]	Action research	Participatory action research	Building usable knowledge	Researchers	School psychology practitioners who work in schools, hospitals and private practice	Social sciences/education	NR
Druskat & Pescosolido, 2002, United States [42]	Shared mental models	Shared mental models	Early stage	Team members	Team members	Social sciences/organisation management	Cognitive theory
Waterman et al., 2001, United Kingdom [43]	Action research	Action research	Problem identification or planning phase	Researchers	Managers, patients, nurses, occupational therapists, students, practitioners, educational staff	Healthcare/health services research	NR
Israel et al., 1998, United States [44]	Action research	Community-based research	Development of partnership	Researchers	Community members, organisational representatives	Healthcare/public health	Critical theory

*NR* none reported

#### Integrated knowledge translation (IKT)

Two reviews referred to knowledge co-production as either IKT [9] or knowledge transfer partnerships [36], thus focusing on the researcher and research user entity. There was no difference between IKT and knowledge transfer partnerships as described by the authors, other than the choice of labels. In this literature, partnership initiation was referred to as the formation stage [9] or pre-research stage [36]. The key actors were referred to as researchers [9, 36] and researcher users were referred to as policy-makers [36] or organisation or system-level decision-makers, including clinician managers, health facility managers and policy-makers [9]. No theory was employed or generated in the two reviews. Gibbon’s Mode 2 knowledge production was mentioned by one review as the origin [9]. The other review reported more recent literature as the origin of knowledge transfer literature but did not mention Mode 2 knowledge production [36]. One reported criticism of Mode 2 knowledge production was that it is not always successful [36].

Both IKT and knowledge transfer partnership reviews mentioned that setting priorities, establishing resources and planning to conduct joint research were processes from the initiation stage of the partnership [9, 36].

#### Action research

Nine of 17 reviews employed various terms synonymous with an action research approach to describe a collaborative or participatory approach to research co-production, thus focusing on the process of partnership development [8, 30, 31, 35, 38, 39, 41, 43, 44]. The terms used to describe the partnership included action research [38, 43], participatory research [8, 31], community-based participatory research [30, 35], community-based research [44], community-based organisation [39], and participatory action theory [41]. In this narrative, partnership initiation was referred to as first stage [30], fostering a partnership [31], partnership development [35], early stage [8], outset of activities [38], gaining entry into the community [39], building usable knowledge [41], problem identification or planning phase [43], or development of partnership [44]. The key actor labels for researchers were researchers [8, 30, 31, 38, 39, 41, 43, 44], academics [39] or academic partners [35]. The key actor labels for research users were community organisation [30], coalition of organisations [30], stakeholders [31, 38], community members [31, 39, 44], end-users [31], community partners [35], people affected by issues under study and/or decision-makers who apply research findings [8], decision-makers [38], school psychology practitioners who work in schools, hospitals and private practice [41], managers, patients, nurses, occupational therapists, students, practitioners, educational staff [43], and organisational representatives [44]. One review mentioned Social Movement Theory [30] as having guided the review, and another mentioned Critical Theory [44]. One review mentioned the origins of action research as first described by Lewin, and then further developed by many, including Moreno, Stenhouse and Elliott, as a method of using science to solve social problems [43]. Criticisms of action research reported in the reviews included that the process is unscientific [43]; that it was challenging to maintain a balance between rigour of the research methodology and incorporating community preferences in the methodological approach [43]; that its results could be biased due to a lack of researcher independence [38, 43]; and that research could be subjective to context and not generalisable [38, 43]. Another review listed the criticism of action research as not being well-described, that it lacked specific procedures for developing partnerships and/or that it involved poorly defined constructs [41].

#### Stakeholder engagement

Two reviews used stakeholder engagement as a label for researcher and research user partnerships [32, 33]. Partnership initiation was referred to as ‘early stage’ by both reviews [32, 33]. The key actors were labelled as researchers [32, 33], and for research users they used the labels patients and public [32], or an individual or group who is responsible for or affected by health and healthcare-related decisions [33]. There were no reported theories guiding the reviews. One origin of stakeholder engagement reported in the review was from Corporate Social Responsibility [33]. There were no criticisms reported in these reviews.

#### Knowledge transfer

Two reviews focused on knowledge transfer by organisational networks and ties [34, 40], focusing on the building of social networks or social capital to transfer knowledge. Both reviews focused on the analysis of the properties of the network ties that allow knowledge transfer to occur [34]. The initiation and sustainment of these ties was relevant to partnership initiation. The labels used for researcher and research user partnerships were structural social capital network for the purpose of knowledge transfer [34] and inter-/intra-organisational network for knowledge transfer [40]. The partnership initiation stage was referred to as a developmental stage by both reviews [34, 40]. They both describe initiation as having a set of activities before the developmental stage, which they referred to as ‘fuzzy-front end’ activities [34] or ‘soft issues’ [40]. One review referred to key actors as researchers [40] and business managers, business partners, customers, suppliers, universities or competing firms [34]. The other review referred to both research and research users as organisation or team members [40]. One of the reviews reported Social Capital Theory as guiding the review [34]. Social Capital Theory described how individuals and entities transfer knowledge [34], and was defined as “*the sum of the actual and potential resources embedded within, available through, and derived from the network of relationships possessed by an individual or social unit*” [34]. The other review did not report any theory but focused on different organisation level processes to transfer tacit and formal knowledge and used the change management literature to outline how relationships are initiated and sustained [40]. In the first review, the origins of knowledge transfer were not discussed in detail [40]. The other review provided a more in-depth summary of the organisational behaviour literature from 1992 to 2012, and focused on the impact of intra- and inter-organisational ties on knowledge transfer [34]. Criticisms reported were that knowledge transfer had many different definitions and that simply increasing the number of ties between organisations did not necessarily result in effective knowledge transfer [34].

#### Team initiation

One review used the project management life cycle to conceptualise team initiation [37]. This review used the label of public–private partnerships, referring to a not-for-profit partnership of researchers and research users intended to generate research [37]. The partnership initiation stage was referred to as the developmental stage [37]. The key actors were referred to as researchers, and research users included government, hospitals, pharmaceutical and biotechnology companies, non-governmental organisations, foundations and experts [37]. There was no theory reported as guiding the review. Although project management is used in many fields, it originated in the 1950s in engineering, as a method to organise and perform projects more efficiently by creating research partnerships. There were no details provided about the origins or criticism of the project management cycle within the review [37].

#### Shared mental models

One review used shared mental models to describe partnerships between researchers and research users [42]. Partnership initiation was referred to as an early stage [42]. They referred to researchers and research users as team members [42]. Cognitive theory was mentioned as guiding shared mental model development [42]. Shared mental models were described as “*socially constructed cognitive structures that represents shared knowledge or beliefs about an environment and its expected behavior*” [42]. The initiation of constructing a shared mental model was relevant to partnership initiation because it described how team members with different skill sets and tasks work together to accomplish a goal. The review reports the origins of term ‘metal model’ as referring to a symbolic representation of a system and the expected behaviour [42]. Theorists used the concept of shared mental models to describe how causal connections and ‘working’ models are collectively constructed by the members of the team in order to calculate potential outcomes or predict future team decisions [42]. There are different types of mental models; the review focused on team mental models, which were linked to team performance [42]. Criticisms of shared mental models were not reported.

### IKT processes, enablers, barriers and outcomes described in narratives

Empirical details about partnership initiation processes, enablers, barriers and outcomes that were evaluated and reported in each review corresponding to each narrative are provided in Additional file 4. They are summarised and compared in Table 3.

**Table 3:** Empirical details about partnership initiation evaluated and reported in each review

Narratives	Team initiation	Stakeholder engagement		IKT partnerships		Action research									Shared mental models	Knowledge transfer	
Reference number	[37]	[33]	[32]	[9]	[36]	[31]	[43]	[35]	[44]	[8]	[30]	[39]	[41]	[38]	[42]	[40]	[34]
Processes																	
Defining and describing the problem and research question	x	x	x		x	x	x		x		x	x		x			
Setting priorities and/or expectations; conducting needs assessment	x		x	x	x		x	x	x			x				x	
Identifying stakeholders and opportunities to build partnerships (internal and external opportunities)	x							x			x	x			x		
Creating common goals with common outcomes, objectives, memorandum of understanding, agreement, operating norms	x		x	x		x	x		x	x	x	x	x	x			
Establishing pre-existing resources that could be used or acquired by the partners to build the project	x			x	x				x		x					x	
Developing risks and benefits of the partnership	x																
Considering inequalities in power	x						x		x							x	
Establishing communication methods such as evidence briefs, web portals, social media, new tools and technologies				x		x						x			x		x
Receiving training and learning		x		x		x			x			x			x		
Applying for funding				x			x		x								
Planning to conduct joint research				x	x				x				x	x			
Establishing committees, boards, or working groups				x													
Creating and transferring of organisational knowledge occurs through processes of conversion (i.e. tacit to formal) and assimilation, and the transfer from individual to collective																x	
Mobilising knowledge/change agents					x											x	
Building organisational structures aligned with strategy and external context	x				x							x				x	
Enablers																	
Sense of ownership of research or output	x		x		x	x	x		x				x		x		
Commitment to partnership	x			x			x		x	x		x			x		
Formal training and development and the acquisition of team members’ knowledge and skills	x								x					x	x		
Positive attitude towards listening, learning, adapting and training		x		x		x							x		x		
Time for team meetings for information sharing by using all-day conference, etc.						x						x		x	x		
Multiple and varied opportunities for interaction				x													
Phased approach to developing shared language				x					x								
Support from facilitators, champions, boundary, spanners; advisory board	x	x		x	x			x	x							x	
Clear and agreed upon goals, roles, expectations and vision	x			x	x	x	x		x		x	x	x			x	
Dedicated funding				x					x		x	x					
Pre-existing relationships between researchers and research users				x					x	x							
Policy-makers with a research background and researchers skilled in policy-making					x												
Supportive policy framework or network structure/ties for researchers and research users to create knowledge and implementing research results			x	x	x			x	x						x		x
Team members from the community						x			x		x	x					
Positive personality of the action researcher							x										
Barriers																	
Time for learning and training, developing relationships, building trust and sustaining intervention		x		x			x	x	x		x	x		x	x		
Performance rewards awarded to individuals rather than groups															x		
Performance feedback that mixed individual with group level feedback															x		
No understanding and/or differing interpretations of the institutional and federal Institutional Review Board regulations		x						x						x			
Imbalance between rigor of academic preferred research designs and incorporating of community preferences							x	x	x			x					
No stakeholder engagement	x	x					x	x						x			
different needs and priorities	x	x					x	x	x								
No skill in understanding of partnership process				x				x									
Negative attitude about researchers or the value of research				x			x		x			x					
Goals, roles and expectations were not clear	x			x			x		x			x			x		
No incentives to participate				x								x					
No funding or infrastructure of partnership	x			x				x	x			x		x			
Little continuity of involvement due to staff turnover				x				x				x					
Limited interaction due to geographic distance		x		x													
Community resistance									x	x							
Issues of power	x	x					x		x			x		x			
Conflict of interest		x										x					
Negative personality of the action researcher							x										
No guidance of initiation of partnerships in literature		x	x				x	x						x			
Outcomes																	
Empowerment of research users			x				x	x	x	x		x					
Develop research questions							x				x	x		x			
Develop a clear understanding of the expectations of different partners	x		x									x					
If research users understand research, they grow to value it, it is more relevant and easier to disseminate and implement, aids in the translation and interpretation of findings which increases actionability		x	x	x			x				x	x	x	x			x
Enhanced mutual understanding of process, including language, work style, needs and constraints, research				x			x							x			
Strengthened relationship, trust and goodwill		x		x						x		x		x			
Emergence of community leaders				x													
Agenda building							x				x	x				x	
Builds strengths and resources within the community							x		x	x	x	x					
Increase trust and respect, minimise fear				x				x	x	x		x		x		x	
Compliance and accountability	x	x	x			x	x	x	x								

#### Processes

The most common initiation stage processes found to be similar across the narratives were ‘identifying stakeholders and opportunities to build partnerships’ found in the narratives of team initiation, action research and shared mental models [30, 35, 37, 39, 42]. ‘Defining or describing the issue or research question’ was found in team initiation, stakeholder engagement, IKT and action research [30–33, 36–39, 43, 44]. ‘Creating project management documentation’, such as common goals, outcomes, objectives, memorandum of agreement and/or operating norms, was found in team initiation, stakeholder engagement, IKT and action research [8, 9, 30–32, 37–39, 41, 43, 44]. ‘Setting priorities and expectations’ was found in team initiation, stakeholder engagement, IKT, action research and knowledge transfer [9, 30, 32, 35–37, 39, 40, 43]. ‘Establishing what skills are available that can be useful for the partnership’ was found in team initiation, IKT, action research and knowledge transfer [9, 30, 36, 37, 40, 44]. ‘Establishing and using communication methods’, such as evidence briefs, web portals, social media, new tools and technologies, was found in IKT, action research, shared mental models and knowledge transfer [9, 31, 34, 39, 42]. ‘Planning to offer training and learning exercises’ was found in stakeholder engagement, IKT, action research and shared mental models [9, 31, 33, 39, 41, 42]. ‘Considering inequalities of power’ was found in team initiation, action research and knowledge transfer [37, 40, 43, 44]. Finally, ‘building organisational structures aligned with both strategy and external context of the partnership’ was found in team initiation, IKT, action research and knowledge transfer [36, 37, 39, 40]. There were only two unique processes of partnership initiation found in the review. Team initiation reported developing risk assessment and benefits analysis of the partnership [37]. A second process that was unique was from knowledge transfer and included establishing a network for creating and transferring organisational knowledge that occurs through processes of conversion (i.e. tacit to formal) and assimilation [40].

#### Enablers

Enablers of initiation found that were similar across narratives were ‘building a sense of ownership of the research produced’, which was found in team initiation, stakeholder engagement, IKT, action research and shared mental models [31, 32, 36, 37, 42–44]. ‘Developing clear and agreed upon goals, roles, expectations and vision for the partnership’ was found in team initiation, IKT, action research and knowledge transfer [9, 30, 31, 36, 37, 39–41, 43, 44]. The personality of the action researcher was an enabler in one action research review [43]. In contrast, ‘support from individuals referred to as key stakeholders or referred to as facilitators, champions, boundary spanners and advisory boards’ was found in team initiation, stakeholder engagement, IKT, action research and knowledge transfer [9, 33, 35–37, 40, 44]. ‘Commitment to partnership’ was reported in team initiation, IKT, action research and shared mental models [8, 9, 37, 39, 42–44]. ‘Formal training, development and acquisition of team members’ knowledge and skills’ was reported in team initiation, action research and shared mental models [37, 38, 42, 44]. ‘Creating an organisational structure or policy framework that supports researcher and researcher user knowledge creation and implementation’ was reported in stakeholder engagement, IKT, action research, shared mental models and knowledge transfer [9, 32, 34–36, 42, 44]. There were two enablers found that were unique. IKT reported planning to have multiple and varied opportunities for interaction [9]. Action research reported the personality of the action researcher as an enabler when perceived as positive by the research users [43].

#### Barriers

Barriers to initiation reported across narratives were a ‘lack of time for learning and training’, ‘developing partnerships’ and ‘building trust and sustaining the intervention’, reported in stakeholder management, IKT, action research and shared mental models [9, 30, 33, 35, 38, 39, 42–44]. ‘Lack of understanding and/or differing interpretations of the institutional and federal regulations by Institutional Review Board administration’ was found in stakeholder management and action research [33, 35, 38]. ‘Lack of stakeholder engagement’ was reported in team initiation, stakeholder engagement and action research [33, 35, 37, 38, 43]. ‘Different needs and priorities among researchers and research users’ was found in team initiation, stakeholder engagement and action research [33, 35, 37, 43, 44]. ‘Unclear goals, roles and expectations’ was found in team initiation, IKT, action research and shared mental models [9, 37, 39, 42–44]. ‘Lack of incentives to participate’ was found in IKT and action research [9, 39]. ‘Lack of funding or infrastructure for partnership team initiation’ [9, 35, 37–39, 44] and ‘lack of continuity due to staff turnover or infrequent meeting attendance’ [9, 35, 39] were found in IKT and action research. Barriers related to partners’ personalities such as attitudes about researchers or the value of research were reported in IKT and action research [9, 39, 43, 44], and issues of power were reported in team initiation, stakeholder engagement and action research [33, 37–39, 43, 44]. Conflict of interest was reported as a barrier in stakeholder engagement and action research [33, 39]. A unique barrier reported in one action research review was the personality of the action researcher, when perceived as negative by the research users [43]. Another unique barrier reported in the shared mental models review was that performance feedback and rewards awarded to an individual, when they should be awarded to a group, can be a barrier in the beginning of partnerships [42].

#### Outcomes

None of the narratives empirically associated outcomes to partnership initiation processes, enablers or barriers; however, several outcomes were proposed. For example, in the IKT, action research and stakeholder engagement narratives, early engagement of research users was linked to increased trust and respect among partners, developing a mutual understanding of language, work style, needs and constraints, which resulted in an increased understanding of the value of research, hypothetically leading, in the future, to easier dissemination and implementation of the research [9, 30, 32, 33, 38, 39, 41, 43]. In the action research and stakeholder engagement narratives, early engagement was a reported outcome that was hypothetically linked to the empowerment of the research user [8, 32, 35, 39, 43, 44] and linked to strengthening relationships, trust and goodwill among the partners [8, 33, 38, 39].

Hypothetical outcomes reported only in the action research narratives included that a strong partnership initiation minimised fear and anxiety of research results [8, 35, 38, 39, 44], helped develop the research question [30, 38, 39, 43], enhanced mutual understanding of processes such as language, work style, needs and constraints [38, 43], and helped create an agenda for the project [30, 39, 43]. In addition, hiring or using resources within the community was linked to facilitating collaborative partnerships in all phases of the research project [8, 30, 39, 43, 44] and increased compliance and accountability of research co-production [31, 35, 43, 44]. Other unique outcomes reported were in the stakeholder engagement narrative where early engagement appeared to be linked with developing a clear understanding of the expectations of different partners [32]. Finally, in the team initiation narrative, unique outcomes linked to early engagement were increased compliance and accountability for research implementation at later stages and clarity of the expectations of different partners [37].

### Conceptual framework of researcher and research user partnership initiation

#### Conceptualisation/theorisation

**Fig. 2:**
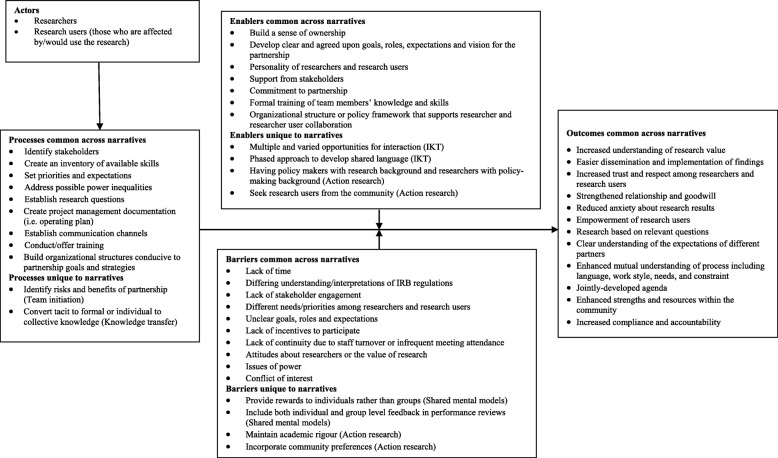
Framework of IKT initiation based on the literature review. This figure summarises the processes, barriers, enablers and outcomes that were found in the review of the literature for the meta-narrative review. It is based on social sciences and healthcare literature combined

Figure 2 consolidates conceptual and empirical findings from narratives across traditions in a conceptual framework of how actors and processes may be influenced by enablers and barriers and the potential associated outcomes of IKT. Overall, initiation is a distinct early phase of IKT in which specific processes (i.e. identify stakeholders, conduct training, establish communication channels) may lead to a variety of outcomes (i.e. trust, respect, goodwill, empowerment, understanding, accountability) if supported by enablers (i.e. organisational policies, jointly developed goals, commitment) and barriers are addressed (i.e. differing priorities, lack of incentives, attitudes about partnership).

#### Over-arching meta-narrative

Partnership initiation was conceptualised differently across narratives; this included the conceptualisation of initiation and the importance ascribed to it with respect to the success of partnership initiatives. Partnership initiation was described as an approach to bring together researchers and research users in order to co-create knowledge [9, 36], a process of the researcher embedding into the community of the research user [8, 30, 31, 35, 38, 39, 41, 43, 44], a process of engaging policy-makers or decision-makers early in the research process [32, 33], or where research users were participants in the research or decision-making [42]. It was also conceptualised as an early phase of the project cycle [37] or as a network developed early for knowledge transfer [34, 40]. Despite the conceptual differences, narratives revealed that processes, enablers, barriers and outcomes were largely common across traditions.

The most common processes were ‘defining or describing the issue or research question’ [30–33, 36–39, 43, 44], ‘creating project management documentation such as common goals, outcomes, objectives, memorandum of agreement and/or operating norms’ [8, 9, 30–32, 37–39, 41, 43, 44], and ‘set priorities and expectations’ [9, 30, 32, 35–37, 39, 40, 43]. The most common enablers were ‘building a sense of ownership of the research produced’ [31, 32, 36, 37, 42–44] and ‘developing clear and agreed upon goals, roles, expectations and vision for the partnership’ [9, 30, 31, 36, 37, 39–41, 43, 44]. The most common barriers were ‘lack of time for learning and training’, ‘developing partnership’, ‘building trust and sustaining the intervention’ [9, 30, 33, 35, 38, 39, 42–44], ‘unclear goals, roles and expectations’ [9, 37, 39, 42–44], and ‘issues of power’ [33, 37–39, 43, 44].

Unique processes of partnership initiation found were ‘developing a risk assessment and benefits analysis of the partnership’ [37] and ‘establishing a network for creating and transferring of organisational knowledge through processes of conversion (i.e. tacit to formal) and assimilation’ [40]. Unique enablers were ‘planning to have multiple and varied opportunities for interaction’ [9] and ‘the personality of the action researcher as an enabler when perceived as positive by the research users’ [43]. Unique barriers were ‘personality of the action researcher, when perceived as negative by the research users’ [43] and ‘performance feedback and rewards awarded to an individual when they should be awarded to a group’, which can be a barrier in the beginning of partnerships [42].

## Discussion

This meta-narrative review was conducted to explore how partnership initiation has been conceptualised, operationalised, and evaluated and, in so doing, identified processes, enablers, barriers and outcomes that are pertinent to partnership initiation. Narratives were generated across six research traditions, namely IKT, action research, stakeholder engagement, knowledge transfer, team initiation and shared mental models, across which partnership initiation was explored, albeit using different labels and descriptions. While initiation was conceptualised differently across traditions, narratives revealed that the majority of partnership initiation processes, enablers, barriers and outcomes were common to multiple narratives. All narratives recognised an initiation stage described as ‘early’ or ‘developmental’. The use of terms such as ‘fuzzy’ or ‘soft’ suggests that the partnership initiation stage has not yet been well conceptualised, despite the fact that formal theories or the origins underlying some of the narratives, such as action research and team initiation, date back several decades. While actors were referred to using various labels, in all narratives, they were categorised as researchers and those who would use or be affected by the research, namely the research users. A common criticism of partnership initiation across the narratives was the challenge of bringing researchers and research users together to jointly undertake research. Proposed outcomes associated with partnership initiation largely included those specific to the relationship between researchers and research users, for example, respect and trust, understanding of research, perceived value of research and clear expectations. Proposed outcomes also included research questions and agendas.

The results of the review are similar to other studies of researcher and research user partnerships in several ways. The partnership initiation processes identified in other partnership studies were also identified in this review; they include setting priorities, establishing virtual and physical communication space [9, 12, 13], clarifying and establishing vision, goals, roles, mission and other project management documents that help to develop the purpose of the partnership [9, 13, 14], and identifying leaders and stakeholders [13–15]. Other partnership studies have also concluded that there are no dominant theories to guide partnership initiation [46, 47], as was found in this review. Another review, that examined the histories and traditions of community-based participatory research and IKT, also found that co-creation of knowledge is referred to with different labels [16]. In addition, similar to other studies, our review failed to identify outcomes definitively associated with partnership initiation processes, enablers or barriers, although several hypothetical outcomes were proposed [9].

While there are commonalities to others’ findings, this review is novel in several ways – it was the first review of a systematic nature to focus on the partnership initiation stage specifically and, as a meta-narrative review, it compiled and compared data on how partnership initiation has been conceptualised, studied and reported in a variety of research traditions. A key output of this review was the conceptual framework of partnership initiation generated from the compilation of knowledge from different research traditions (Fig. 2). Researchers or research users interested in establishing partnerships, regardless of research tradition or discipline, can draw on the conceptual framework to plan partnership initiation processes, anticipate challenges and identify performance measures or relevant outcomes.

The strengths of this research include the use of rigorous review methods, i.e. independent pilot testing of screening and data extraction [24], compliance with standards for literature searching [29] and adherence to reporting standards for meta-narrative reviews [19]. Still, a few issues may limit the interpretation and use of these findings. Although we searched multiple relevant databases, we may not have identified all relevant reviews. By reviewing reviews, we may not have fully captured all IKT concepts or descriptions reported in primary studies. As noted earlier, studies relevant to researcher and research user partnerships are not well indexed and, thus, hard to discover [1]. The choice of methodology of this study may have had an impact on the results in that we relied on what was reported in the literature that was analysed. For example, if primary studies were to be analysed covering many more subject disciplines, the resulting processes, barriers, enablers and outcomes could be expanded upon. In addition, our choice of publication types – systematic reviews – poses a limitation of qualitative studies included in our sample, which could add to the conceptualisation of initiation. Furthermore, 11 of the included reviews did not follow any reporting guidelines or recommendations and this may pose a risk to the quality of the data gathered for this analysis. Finally, the labelling of the narratives was based on the partnership labels found in the 17 reviews, not their primary studies, which may have used many more labels to describe researcher and research user partnerships.

Nevertheless, this review consolidated knowledge about IKT initiation across several research traditions to generate a conceptual framework that can guide ongoing research and practice in this area. While a meta-narrative review is meant to describe how a phenomenon has been conceptualised, that information was not always detailed in the included reviews and the primary research they synthesised. By having consolidated conceptual and theoretical knowledge, we may have overcome this limitation of the included reviews. However, further research is needed to thoroughly test the relationships between actors, processes, enablers, barriers and outcomes of IKT initiation described in the conceptual framework we generated. To generate further insight, such testing should employ theory, which was lacking in most reviews. This may also reveal outcomes associated with initiation not yet identified across traditions examined in this review, and more firmly establish outcomes associated with initiation.

## Conclusions

This meta-narrative review of 17 reviews published from 1998 to 2017 identified a partnership initiation phase referred to as ‘early’ or ‘developmental’ across six research traditions – IKT, action research, stakeholder engagement, knowledge transfer, team initiation and shared mental models. Few reviews employed or discussed relevant theory, and initiation was conceptualised differently across traditions. However, the majority of partnership initiation processes, enablers, barriers and outcomes were common to narratives across multiple traditions. Conceptual and empirical findings were consolidated in a conceptual framework of IKT initiation that can be employed by researchers in various traditions or research users to study or practice IKT initiation. Although this review identified considerable congruence across research traditions, relationships between components of the conceptual framework remain hypothetical; therefore, further research is needed to fully test the relevance of the conceptual framework.

## Supplementary information

**Additional file 1.** RAMASES criteria, where each criteria is met within the manuscript.

**Additional file 2.** MEDLINE Search Strategy. The final search strategies conducted in MEDLINE on June 9, 2017.

**Additional file 3.** Data extracted from included reviews. Complete data extraction form for each of the 17 included reviews.

**Additional file 4.** Empirical details about partnership initiation. Details about partnership initiation that were evaluated and reported in each of the 17 included reviews.

## Data Availability

All data generated or analysed during this study are included in this published article and its supplementary information files.

## Abbreviation

IKT: integrated knowledge translation
