# Supplementary material for: Conceptualising the initiation of researcher and research user partnerships: a meta-narrative review
Source: Health Res Policy Syst. 2020 Feb 18;18:24. doi: 10.1186/s12961-020-0536-9 (PMC7029453; doi:10.1186/s12961-020-0536-9)
Supplement: Supplementary file 2 — Additional file 2. MEDLINE Search Strategy. The final search strategies conducted in MEDLINE on June 9, 2017. [file 12961_2020_536_MOESM2_ESM.docx]

# Additional file 2. MEDLINE search strategy

| **#** | **Searches** | **Results** |
| --- | --- | --- |
| 1 | (integrat* adj2 knowledg* adj2 translat*).mp. | 98 |
| 2 | (knowledg* adj2 translat*).mp. | 3197 |
| 3 | (knowledg* adj2 synth*).mp. | 704 |
| 4 | (knowledg* adj2 disseminat*).mp. | 918 |
| 5 | (knowledg* adj2 exchang*).mp. | 789 |
| 6 | Community-Based Participatory Research/ | 3291 |
| 7 | "engaged scholarship".mp. | 38 |
| 8 | "action research".mp. | 3401 |
| 9 | "participatory research".mp. | 5068 |
| 10 | (knowledg* adj2 action).mp. | 764 |
| 11 | "know do gap".mp. | 57 |
| 12 | "shared decision making".mp. | 4893 |
| 13 | "shared mental model*".mp. [ | 112 |
| 14 | "stakeholder engagement".mp. | 526 |
| 15 | *Translational Medical Research/ed, is, lj, mt, og, td [Education, Instrumentation, Legislation & Jurisprudence, Methods, Organization & Administration, Trends] | 1881 |
| 16 | 1 or 2 or 3 or 4 or 5 or 6 or 7 or 8 or 9 or 10 or 11 or 12 or 13 or 14 | 19568 |
| 17 | limit 16 to (meta analysis or "review" or systematic reviews) | 3777 |
