# Supplementary material for: Conceptualising the initiation of researcher and research user partnerships: a meta-narrative review
Source: Health Res Policy Syst. 2020 Feb 18;18:24. doi: 10.1186/s12961-020-0536-9 (PMC7029453; doi:10.1186/s12961-020-0536-9)
Supplement: Supplementary file 3 — Additional file 3. Data extracted from included reviews. Complete data extraction form for each of the 17 included reviews. [file 12961_2020_536_MOESM3_ESM.docx]

Additional File 3. Data extracted from included studies

| Author  Year  Country | Partnership | | | Partnership Initiation processes, enablers, barriers, outcomes | Discipline/  Field of study/  Narrative | Theory, origin, criticisms |
| --- | --- | --- | --- | --- | --- | --- |
|  | Partnership label and description | Review details | Key actors |  |  |  |
| Tremblay/ 2017/  Canada | Community-based participatory research is an approach to research that engages community and academic partners in a common knowledge production process aimed at understanding and improving the well-being and health of groups and communities. | Type: theoretical review  Purpose: this study aims to describe the development of a multidimensional conceptual framework building on social movement theories capable of drawing out identifiable elements of CBPR processes. Number of articles: 58  Years: Up to August 1, 2015 | Types of partners: Researchers; community members | Label: First stage  Processes:   - Define the issue of interest - Identify stakeholders/parties interested in problem - Goals/Elements of the context to take into consideration relating to the problem - Establish resources available   Enablers   - Clear goals - Dedicated funding, office, materials - Intangible resources, skills from the community   Barriers   - Lack of time for learning, developing relationships   Outcomes:   - Research question - Research output is more relevant and has more value, helps implementation - Relevant agenda - Strengthen community ties | Health Sciences/ Community Psychology/ Team-initiation | Theory: Social Movement Theory  Origin: Action Research Criticism: NR |
| Gagliardi/ 2016/  Canada | Integrated knowledge translation | Type: Scoping Review  Purpose:  • to characterize the nature of research in this area (IKT),  • to describe IKT strategies that were empirically evaluated,  • to reveal whether sufficient research is available to undertake a systematic review of the effectiveness of various IKT approaches,  • to identify knowledge gaps for future IKT research  Number of articles reviewed: 13  Years: 2005-2014 | Researchers; Research users described as: organization or system-level decision-makers including clinician managers, health facility managers, and policy-makers | Label: Formation stage  Processes   - Priority-setting - Set goals - Pre-existing resources - Communications methods, such as meetings, evidence briefs, emails, web - Training sessions - Applying for funding - Joint research - Committees, boards, or working groups   Enablers   - Strong leadership commitment, skill and experience - Phased approach to develop shared language, achieve early successes - Support from enablers, champions, and boundary spanners - Clear and agreed upon goals, roles, and expectations - Immersion of researchers in research users setting/co-location - Attitude about researchers or the value of research - Multiple opportunities of interaction   • Shared governance structures   - Openness of partners to listen, learn, and adapt - Organizational support for research users to meaningfully contribute - Dedicated funding - Built on pre-existing relationships   Barriers   - Differing needs and priorities - Lack of skill in understanding of IKT process - Goals, Roles, expectations not clear - Lack of incentives to participate - Lack of funding or infrastructure of IKT - Little continuity of involvement due to staff turnover, infrequent attendance - Participants are busy with multiple responsibilities - Geographic distance imposes limits on interaction   Outcomes   - Research users grew to value research - Enhanced relevance of the research - Enhanced mutual understanding of language, work style, needs, and constraints - Strengthened relationship, trust and goodwill - Emergence of community leaders | Healthcare/ Health Services Research/IKT | Theory: NR  Origin: Gibbons (1994)  Criticism: NR |
| Salsberg/  2015/ Canada | Participatory Research: umbrella term to include community-based participatory research, action research, participatory action research, participatory evaluation, community engagement, and community engagement and patient engagement | Type: Critical review  Purpose: To undertake a critical review describing key strategies supporting development of participatory research (PR) teams to engage partners for creation and translation of action-oriented knowledge; Number of papers: 54  Years: 1995-2009 | Researchers; stakeholders (end-users, community members) | Label: Strategies for fostering a researcher-community partnership  Processes   - Form a community-advisory committee, - Develop research agreements, formal goals, roles responsibilities, questions - Use facilitation techniques to establish communications, circulate agendas, small group work. - Hire from community - Frequent communication   Enablers   - Advisory committee, Action planning, Interpretation, data ownership, and dissemination, - Build sense of ownership - Positive attitude for learning and training - Frequent meetings - Clear goals, roles, expectations - Hire from the community   Barriers: NR  Outcomes:   - Compliance and accountability for research output | Healthcare/ Public Health/ Action Research | Theory: NR  Origin: Action research  Criticism: NR |
| Esmail/2015/United States | Stakeholder engagement research: Researchers doing research with patients rather than for, at or to them | Type: critical review  Purpose: to synthesize what the literature proposes as the hypothesized impacts of engagement (i.e., the benefits), share what has been evaluated and assessed and propose the steps needed to reduce the gap between research engagement's promises and the underlying evidence base supporting its practice  Number of papers: 108  Years: 2005-2013 | Patients, public, stakeholders; researchers | Label: Early stage  Processes   - Stakeholder engagement for research question - Engage early to provide unique perspectives, direct knowledge, helps set priorities - Common goals, roles, outcomes   Enablers   - Sense of ownership - Support from stakeholders   Barriers   - lack of assessment of engagement   Outcomes:   - Empowering patients, - Early ambassadors of research, increased translation, dissemination and uptake: more understanding from the patient makes for better research uptake - Democracy and accountability/Moral obligation | Healthcare /Health Services Research /Stakeholder engagement | Theory: NR  Origin: NR  Criticism: NR |
| Concannon/2014/  United States | Stakeholder engagement: engagement as a bi-directional relationship between stakeholder and researcher that results in informed decision-making about the prioritization, conduct and use of research | Type: Systematic Review  Purpose: to catalogue reported methods of stakeholder engagement in comparative effectiveness research and patient-centered outcomes research.  Number of papers: 70  Years: 2003-2012 | Researches; Individual or group who is responsible for or affected by health-and healthcare-related decisions that can be informed by research evidence | Label: Early stage  Processes   - Define problem - Training   Enablers   - Positive attitude to learning and training - Support from stakeholders   Barriers   - Lack of time; - Managing conflict; - Geographic distance - Lack of stakeholder engagement/commitment - Increased ethical concerns in some institutional review boards (IRB); - addressing implicit power differentials/Issues of power; - Conflict of interest, - Lack of reporting on engagement detail   Outcomes:   - improved relevance of research; - increased stakeholder trust in research users and researchers, - improved research adoption - Compliance/joint decision-making | Healthcare/ Medicine/  Stakeholder engagement | Theory: NR  Origin: Corporate social responsibility  Criticism: SR) |
| Filieri/2014/ United Kingdom | Structural social capital: the patterns of social ties characterising a group of actors, it concerns the properties of the social system and the network of relation as a whole | Type: systematic review  Purpose: In this paper, we present a comprehensive and critical review of the literature on structural SC and its influence on knowledge transfer practices adopted to share different types of knowledge at different levels. The review reveals the linkages between the different dimensions of structural SC and the transfer of different knowledge resources at intra- and inter organizational levels which subsequently affect innovation. The aim of this study is to review the studies on structural SC, knowledge transfer and innovation at different level of analysis, namely at the intra-firm and inter-firm levels.  Number of papers: 109  Years: 1992-2012 | Business managers; business partners; customers; suppliers; universities; and competing firms | Label: Fuzzy-front end and development stage  Processes: NR  Enablers:   - Modern communication technologies (e.g. Skype), social networking (e.g. Socialcast, Facebook) and crowdsourcing platforms (e.g. Innocentive.com) are increasingly allowing employees and businesses to connect, collaborate and work together and share knowledge without the necessity of having intimate relationships and frequent face-to-face interactions; - Strong ties can enhance different KT processes such as knowledge integration at the inter-firm level;   Barriers: NR  Outcomes:   - If consumers and others are involved in product development the product will be better suited for consumers; | Social Sciences /Knowledge Management/ Knowledge transfer | Theory: Social Capital Theory  Origin: Knowledge Transfer);  Criticisms: hard to deal with because of different definition of Knowledge Transfer; increasing the number of ties between organizations does not necessarily result in effective knowledge transfer |
| Andrews/ 2012/  United States | Community based participatory research: a partnership approach to scientific inquiry that involves collaboration among community members, community partners and academic researchers throughout the research process | Type: Systematic review  Purpose: The purpose of this paper is to review the relevant literature on the use, quality, and effectiveness of CBPR for smoking cessation interventions. Number of articles: 23  Years: 1995-2011 | Community partner/Community organization; Academic partner | Label: Partnership development  Processes   - Set priorities - Identify stakeholders by advisory board   Enablers   - Seek support from advisory board to guide the process of research; - Create supportive framework   Barriers   - Stakeholder not involved in all stages of research; - Lack of funding - Navigating the IRB for CBPR studies is often time consuming. Either a lack of understanding and/or differing interpretations of the institutional and federal IRB regulations by IRB administrators and investigators may impede the process. - Time involved in developing relationships, - Building trust, and sustaining intervention effects; partnerships may take years to develop; - Maintaining the rigor of academic preferred research designs (i.e., randomized controlled trials; - Lack of continuity - Lack of data on initiation   Outcomes:   - Community empowerment; - Increase trust - Accountability for research. | Healthcare/  Public Health/  Action Research | Theory: NR  Origin: NR  Criticism: NR |
| Jagosh/2012/Canada | Participatory research: co-construction of research through partnerships between research and people affected by and/or responsible for action on issues under study. | Type: Realist review  Purpose: our synthesis concentrated on the impact of co-governance on research processes and outcomes; Number of articles: 276  Years: 1970-2011 | Researchers, and people affected by issues under study and/or decision makers who apply research funding | Label: Early stage  Processes:   - Creating common goals   Enablers   - Commitment to partnership, synergy created - Pre-existing relationships   Barriers:   - community resistance   Outcomes   - Greater empowerment, - Established trust, respect - Deepened stakeholders' commitment to the project | Healthcare/  Health Services Research/ Action Research | Theory: NR  Origin: NR  Criticism” NR |
| Orem/2012/Uganda | Knowledge translation; Partnership between policy makers and researchers to increase Knowledge translation in health policy in low income countries | Type: Descriptive review  Purpose: This paper aims at contributing to gap in how partnerships increase knowledge translation in health policy in low income countries. Its main objective is to elaborate a Middle Range Theory (MRT) of KT in Uganda that can also serve as a reference for other low- and middle countries; Number of articles: 49  Years: 2000-2010 | Policy makers; researchers, | Label: Pre-research stage  Processes   - Prioritization of research addressing policymakers’ information need, research priority setting - Provide capacity for research, analysis and synthesis - Identify champion/knowledge broker - Plan for joint research - Build capacity for policymakers and researchers support of the project - Create an institutionalized mechanism of getting researchers involved in policy making and policymakers involved in research   Enablers   - Ownership of research results by policymakers; - Supportive policy frameworks (clear goals) - Support from stakeholders - Having policymakers with a research background and researchers skilled in policy making - Supportive policy framework for implementing research results (guidelines, plans, monitoring frameworks)   Barriers: NR  Outcomes: NR | Healthcare/  Health Policy/ IKT | Theory: NR  Origin: Knowledge translation  Criticism: not always successful |
| De-Pihno Campo/  2011/  Canada | Public-private partnerships referring to not-for-profit partnerships | Type: Systematic review  Purpose: to identify empirical-based descriptive articles to understand critical elements in the partnership process, and propose a framework to shed light on future guidelines to support better planning, design and management of existing and new forms of PPPs for public health.  Number of articles: 10  Years: 1990-2010 | Researchers; Research institutions, government, hospitals, pharmaceuticals and biotechnology companies, NGOs, foundations, experts, investors, | Label: Development stage  Processes:   - Define goals, responsibilities, IP rights, and project management aspects; - Organizational structure to allow for mutuality of interests among partner organizations in terms of expectations from the project; - Conduct, risks and benefits of the partnership; - Manage power relations; - Conduct appraisal of local context and infrastructure, understanding local capacity. - Each partner must bring to the table something worth to others and that it be aligned with the goals of the partnership; - get a clear understanding of the expectations of different partners; - Identify stakeholders   Enablers:   - Build a strong sense of ownership; - Commitment - Training and development for team skills - Support from managers, stakeholders - Clear goals, expectations   Barriers:   - Lack of stakeholder engagement - Different priorities - Unclear goals, roles, expectations - Lack funding or infrastructure of partnership - Power issues   Outcomes:   - Gaining acceptance among partner organization expectations and needs - Compliance and accountability during the project; | Healthcare/  Public Health/  Team initiation | Theory: NR  Origin: Project management (PMBOK)  Criticism” NR |
| Chiasson/ 2009/United Kingdom | Action research: form of applied research that develops a solution to a practice problem, which is of value to the people with whom the researchers are working, while at the same time developing theoretical knowledge of value to a research community. | Type: Systematic review  Purpose: to investigate the role of pluralist approaches in AR. AR researchers should follow two parallel cycles, the research cycle and the problem-solving cycle. The first analysis is focused on the level of each AR study and it investigates how research and problem-solving cycles are mixed and interact during AR studies. Second, we focus on the wider research programme in which the AR is situated and investigate how AR is mixed with other research methods to produce knowledge for IS practice and theory.  Number of articles: 63  Years: 1982-2005 | Researchers, stakeholders, decision-makers | Label: Outset of activities  Processes   - Describe problem or issue - Create relevant goals - Plan for joint research   Enablers   - Training - Make time for meetings   Barriers:   - Lack of time - Lack of understanding of institutional policies - Lack of stakeholder engagement - Lack of infrastructure for partnership - Inequalities - Lack of reporting   Outcomes   - Relevant research question - Research more likely to be implemented if understood by research users - Increased trust - Decreased fear or anxiety of results | Social Sciences/  information Systems/  Action Research | Theory: Many theories can be applied to AR  Origin: Just for AR in Information Systems  Criticisms: lack of impartiality of the researcher; lack of discipline; often mistaken for consulting; context-dependency leading to difficulty in generalizing results |
| Suarez-Balcazar/ 2005/United States | Community-university collaborations: explicit written or verbal agreement between a community setting (community-based organization [CBO] | Type: Theoretical review  Purpose: proposes an interactive and contextual model for developing and sustaining community-university partnerships; Number of articles: 54  Years: 1977-2004 | Academics and community members | Label: Gaining entry to community, developing mutual collaboration  Processes   - Involvement in the community to understand issue or develop question - Develop project mission, goals, roles, and expectations of the partnership and a common vision - Establish communication methods - Learn about the community, developing a culture of learning - Build structures to facilitate exchange   Enablers   - Commitment - Make time for information sharing - Clear mission, vision, goals, develop a collaborative action agenda - Dedicated funding - Hire from community or get community members involved on the team   Barriers   - Maintain rigor - Lack of incentives - Lack of understanding of research value - Issues of Power and Resource Inequalities; - Time Commitment; - Conflict of Interest; - Budget Cuts and End of Funding; - Community resistance - Staff turnover   Outcomes   - creating mission, goals, roles, and vision, research questions; - communication with partners, - developing trust and mutual - Empowerment - Develop action agenda. | Social Sciences/  Psychology /  Action Research | Theory: NR  Origin: NR  Criticism: NR |
| Guzman/ 2005/  Australia | inter and intra-organizational knowledge transfer | Type: theoretical review  Purpose: the goal of this study is to contribute, from the organizational perspective, towards the understanding of key aspects that shape the transfer of organizational knowledge. Based on a case study and literature review, an architecture for understanding the existing theoretical framework is delineated first; to point out the 'soft' managerial aspects that need to be considered to support the transfer process. Number of articles: 29  Years: 1979-2003 | Organization or team members | Label: “Soft” issues before developmental stage of collaboration  Processes   - Define expectations and priorities - Consider power and politics - Establish available resources and skills; Solving operational problems by drawing on cross-functional expertise - Allow channels of knowledge transfer from tacit to formal and individual to collective; disseminate knowledge through all organizational levels - Mobilize knowledge/change agents - Build organizational structures aligned with both strategy and external context   Enablers   - Support from enablers - Shared goals, agenda building   Barriers: NR  Outcomes   - Gaining attention and agenda building; - Gaining legitimacy and backing, trust | Social Sciences/  Knowledge Management/ Knowledge transfer | Theory: Many theoretical frameworks can be applied depending on context  Origin: Organizational Knowledge Transfer  Criticism: NR |
| Riley-Tillman/  2005/United States | Participatory action research (PAR): a model of research designed to develop new technology (or innovation) that is considerate of the realities of practice; occurs through ongoing collaboration between researchers and practitioners within the design and implementation phases of the research process. | Type: Narrative review  Purpose: we discuss the history behind efforts to transfer school psychology research into practice and present a model for systematically  programming for this transfer. In particular, the literature regarding treatment acceptability, participatory action research, organizational change, and generalization programming are reviewed given the direct relevance to our proposed framework for transferring school psychology research into practice.  Number of articles: 49  Years: 1977-20015 | Research users are School psychology practitioners work in many settings including schools, hospitals, and private practice; researchers are: trainers/researchers are  typically, in university settings, and focus on training individuals to use and conduct research regarding issues related to the field of school psychology | Label: Initiation processes  Processes   - Create common goals - Jointly developing knowledge with all stakeholders   Enablers   - Sense of ownership - Positive attitude towards partnership - Common goals   Barriers: NR  Outcome   - Research user involvement in development of interventions has a high potential for generalization implementation in school settings | Social Sciences/  Education/  Action Research | Theory: NR  Origin: NR  Criticism: not well-described; lack specific procedures for developing partnerships, and/or involve poorly defined constructs |
| Druskat/  2002/United States | Shared mental models emerge as teams interact to make sense of their situation and cultivate shared beliefs about how they should work together to complete their task. There are 3 primary sources that influence the development and sustainability of the SMM that emerge in a team: team member history or prior experience in teams, team task, and organizational culture and environment | Type: Theoretical review  Purpose: to identify the content of effective shared mental models in self-managing work teams (SMWT), to examine how these effective mental models emerge in SMWT, and to examine how they endure in dynamic organizational context;  Number of articles: 4  Years: 1986-1996 | Team members | Label: Early stage  Processes   - Identify internal and external leaders - Establish communication methods - Training and learning   Enablers   - Build psychological ownership - Continuous learning, formal training and development and the acquisition of team members’ knowledge and skills Positive attitude towards model - Make time for meetings and training - Supportive framework for mental model development; team members' serious approach to learning contributed to early success   Barriers   - Organizations provided less time for formal training and education - Lack of resources - Performance rewards awarded to individuals rather than groups, - Performance feedback that mixed individual with group level feedback; - External leaders were experiencing mixed messages about their roles   Outcomes: NR | Social Sciences/  Organizational Management/  Shared Mental Models | Theories: Cognitive Theories  Origin: Psychology  /Education  Criticism: NR |
| Waterman/ 2001/United Kingdom | Action research: a period of inquiry that describes, interprets, and explains social situations while executing a change intervention aimed at improvement and involvement…founded on a partnership between action researchers and participants, all of whom are involved in the change process | Type: Systematic Review  Purpose: 1. To provide a definition of action research (see chapter 3). 2. To identify published and unpublished action research projects conducted in healthcare  settings in the UK. 3. To analyse action research in the healthcare field, by looking at:  • aims of action research  • reasons for choosing action research  • issues addressed by action research  • outcomes and impacts of action research  • pivotal factors – strengths and limitations  (see chapter 6).  4. To develop guidance for the development and assessment of action research proposals and reports  Number of articles: 59  Years: 1975-1998 | Researchers; Managers, patients, nurses, occupational therapists, students, practitioners, educational staff, | Label: Problem identification phase and planning phase  Processes   - Problem identification - Set priorities - Create common objectives, - Establish rapport, manage inequalities of power - Apply for funding together   Enablers   - promotes ownership of change - willingness to participate - Clear expectations, goals - Personality of the researcher   Barriers   - Lack of time - Maintain academic rigour - Lack of participation - Different goals, priorities - Research not valued, lacked concern for outcome - Issues of power - Personality of the action researcher - Lack of data on initiation   Outcomes   - Empowerment of researcher user - Relevant research question or research scope - If research user understands research, more likely to implement - Greater understanding of project from both researchers and research users - Build a relevant agenda - Build resources within the community - Compliance and accountability | Healthcare/  Health Services Research/  Action Research | Theory: NR  Origin: Action Research (Kurt Lewin; Jacob Moreno; Lawrence Stenhouse and John Elliott)  Criticisms:  Unscientific/keeping rigour, biased due to lack of researcher independence; research is subjective to context and not generalizable |
| Israel/1998/ United States | Community-based research in public health is a collaborative approach to research that equitably involves for example, community members, organizational representatives, and researchers in all aspects of the research process. | Type: narrative review  Purpose: to synthesize key principles or characteristics of community based research; to examine community-based research within the context of different scientific paradigms; to discuss rationales for its use; and to explore challenges and facilitating factors and their implications for conducting effective community-baed research aimed at improving the public's health. Number of articles: 130  Years: 1968-1997 | Community members, organizational representatives; Researchers | Label: Development of partnership  Processes:   - Define scope or problem - Set priorities - Identification of common goals and objectives Establish list of resources - Consider power and control - Training and learning - Apply for funding together - Conduct research together   Enablers   - Build sense of ownership - Development of jointly agreed upon research principles, jointly developed operating norms - Commit to partnership - Formal training, researchers’ role skills, and competencies are clear - Involvement of support staff/team - Identification of key community members - Support and involvement of community members - Phased approach to develop partnership - Funding - Prior history of working relationship - Hire from community - Organizational structure that allows partnerships, feasibility and benefit of shared control on all aspects of the research process   Barriers   - Lack of time - Maintain rigour - Differing needs or priorities - Conflicts associated with differences in perspectives, priorities, assumptions, values, beliefs and language - Conflicts over or lack of funding - Community resistance - Power and control, reluctance for community partners to participate because they were familiar with the hierarchical modes of decision-making in universities and their reluctance to share power and control   Outcomes   - Builds strengths and resources within the community, - Compliance and accountability - Increase trust and respect | Healthcare/  Public Health/  Action Research | Theory: NR  Origin: NR Criticism: NR |

NR not reported
