# Supplementary material for: Conceptualising the initiation of researcher and research user partnerships: a meta-narrative review
Source: Health Res Policy Syst. 2020 Feb 18;18:24. doi: 10.1186/s12961-020-0536-9 (PMC7029453; doi:10.1186/s12961-020-0536-9)
Supplement: Supplementary file 4 — Additional file 4. Empirical details about partnership initiation. Details about partnership initiation that were evaluated and reported in each of the 17 included reviews. [file 12961_2020_536_MOESM4_ESM.docx]

Additional File 4. Empirical details about partnership initiation

| Narrative | Partnership initiation details that were examined and reported | | | |
| --- | --- | --- | --- | --- |
|  | Processes | Enablers | Barriers | Hypothetical Outcomes |
| IKT | - Setting priorities, establish resources and plan to conduct joint research (9, 36) - Defining and describing the problem or research question (36) - Mobilizing knowledge and change agents (36) - Building organizational structures aligned with strategies and external context (36) - Creating common goals to common outcomes, objectives, memorandum of understanding, agreement, and operating norms (9) - Establishing communication methods (9) - Receiving training and learning, applied for funding, and established committees, boards and working groups (9) - Applying for funding (9) | - Support from individuals such as facilitators, champions, boundary spanners or an advisory board (9, 36) - Clear and agreed upon goals, roles, expectations, and vision (9, 36) - Supportive policy framework or network that encourages researchers and research users to create and implement knowledge (9, 36) - A sense of ownership of research output (36) - Policymakers with a research background and researchers skilled in policy-making on the team (36) - Commitment to partnership, had positive attitude towards listening, learning, adapting, and training, created a multitude and varied opportunities for interaction [9] - Phased approach to develop shared language (9) - Dedicated funding for the partnership (9) - Pre-existing relationships between researcher and research users(9) | - Lack of time for learning and training, developing relationships, building trust, and sustaining intervention (9) - Lack of understanding of partnership processes (9) - Negative attitude towards researchers or the value of research (9) - Unclear goals, roles and expectations (9) - Lack of incentives to participate (9) - Lack of funding or infrastructure for IKT (9) - Little continuity of involvement due to staff turnover or infrequent attendance (9) - Limited interaction due to geographic distance (9) | - Early engagement of research users increased research users’ understanding of the research, which results in an increased understanding the value, easier dissemination and implementation and interpretation of findings (9) - Increased trust and respect among researchers and research users, thus minimized fear and anxiety of research results (9) - Build strength and resources within the community (9) - Facilitated collaborative partnerships in all phases of the research project (9) - Enhanced mutual understanding of process including language, work style, needs and constraints (9) - Identify community leaders (9) |
| Action research | - Creating common goals and objectives with common outcomes by developing a memorandum of understanding, an agreement, developing operating norms (8, 30, 31, 38, 39, 41, 43, 44) - Defining and describing the problem or research question (30, 31, 38, 39, 43, 44) - Setting priorities and/or expectations by conducting a needs assessment or other method (35, 39, 43, 44) - Identifying stakeholders and opportunities to build internal and external partnerships (30, 35, 39) - Conducting training and learning exercises (31, 39, 44) - Planning to conduct joint research (38, 41, 44) - Establishing pre-existing resources that can be used or acquired for the project (30, 44) - Considering how to manage inequalities of power (43, 44), establishing communication methods for the project (31, 39) - Jointly applying for funding (43, 44) - Building organizational structures aligned with both strategy and external context (39). | - A sense of ownership of research or its output (31, 41, 43, 44) - Commitment to partnership (8, 39, 43, 44) - Formal training and development related to the project (38, 44) - Positive attitude towards listening, learning, adapting and training (31, 41) - Time for team meetings (31, 38, 39) - Support from individuals such as facilitators, champions, boundary spanners or an advisory board (35, 44) - Clear and agreed upon goals, roles, expectations, and vision (30, 31, 39, 41, 43, 44) - Dedicated funding to the partnership (30, 39, 44) - Pre-existing relationship between researchers and research users (8, 44) - Supportive policy framework or network that encourages researchers and research users to create and implement knowledge (35, 44) - Members from the community or have the researchers being involved in the target community in a significant way such as volunteering, dedicating time to learn about the community, join community events, read reports and other publications (30, 31, 39, 44) - A phased approach to develop shared language (44) - Researcher had positive personality (43). | - Lack of time for tasks including learning and training, developing relationships, building trust and sustaining interventions (30, 35, 38, 39, 43, 44), - Lack of understanding or differences in interpretations of institutional IRB policies between researchers and research users (35, 38) - Did not maintained a balance between academic rigor and community preferences (35, 39, 43, 44) - Lack of stakeholder engagement (35, 38, 43) - Differing needs and priorities (35, 43, 44) - Negative attitude towards researchers or value of research for the community (39, 43, 44) - Unclear goals, roles and expectations (39, 43, 44) - Lack of funding or infrastructure for partnership (35, 38, 39, 44) - Little continuity of involvement due to staff turnover or infrequent attendance (35, 39) - community resistance (8, 44) - Issues of power (38, 39, 43, 44) - Lack of data on initiation of partnerships (35, 38, 43) - Conflict of interest (39) - Lack of skill in understanding partnership processes (35) - Negativity from action research (43) | - Early engagement of research users increased research users’ understanding of the research, which results in an increased understanding the value, easier dissemination and implementation and interpretation of findings (30, 38, 39, 41, 43) - Increased trust and respect among researchers and research users, thus minimized fear and anxiety of research results (8, 35, 38, 39, 44) - Empowerment of the research user (8, 35, 39, 43, 44) - Developed the research question (30, 38, 39, 43) - Got a clear understanding of the expectations of different partners (39) - Enhanced mutual understanding of processes such as language, work style, needs and constraints (38, 43) - Strengthened relationship, trust and goodwill (8, 38, 39) - Built an agenda for the project (30, 39, 43) - Built strength and resources within the community, which facilitates collaborative partnerships in all phases of the research project (8, 30, 39, 43, 44) |
| Stakeholder engagement | - Defining or describing problem or research question (32, 33) - Setting priorities and/or expectations by conducting a needs assessment or other method (32) - Creating common goals to common outcomes, objectives, memorandum of understanding, agreement, and operating norms (32) - Conducting training and learning activities (33) | - A sense of ownership of research or output (32) - Supportive policy framework or network structures/ties for researchers and research user to create knowledge and implement research results (32) - Positive attitude towards listening, learning, adapting and training (33) - Support from facilitators, champions, boundary spanners or an advisory board (33) | - Lack of reporting of partnership initiation in the literature as a barrier to initiating partnerships (32, 33) - Lack of time for learning and training, developing relationships, building trust and sustaining interventions (33) - Lack of understanding or differing interpretations of the institutional and federal IRB regulations (33) - Lack of stakeholder engagement, differing needs and priorities (33) - Limitations due to geographic distance (33) - Issues of power, and conflict of interest (33) | - Early engagement of research users increased research users’ understanding of the research, which results in an increased understanding the value, easier dissemination and implementation and interpretation of findings (32, 33) - Empowerment of research users (32) - Got a clear understanding of the expectations of different partners (32) - Strengthened relationship between researchers and research users (33) - Increased compliance and accountability of research implementation (32, 33) |
| Knowledge transfer | - Setting priorities and/or expectations by conducting a needs assessment or other method (40) - Establishing pre-existing resources that can be used or acquired for the project (40) - Considering any inequalities in power (40) - Establishing processes to convert and assimilate tacit knowledge to formal knowledge (40) - Mobilizing knowledge/change agents who coordinate processes to create and diffuse knowledge (40) - Building organizational structures aligned with both strategy and external context (40) - Establishing communication methods (34) | - Support from facilitators, champions, boundary spanners, or advisory boards(40) - Clear and agreed upon goals, roles, and expectations (34) - Supportive policy framework or network structures/ties for researchers and research users to create knowledge and implement research results (34) |  | - Early engagement of research users increased research users’ understanding of the research, which results in an increased understanding the value, easier dissemination and implementation and interpretation of findings (34) - Increased trust and respect among researchers and research users, thus minimized fear and anxiety of research results (40) - Built an agenda (40) |
| Team initiation | - Defining or describing problem or research question (37) - Setting priorities and/or expectations by conducting a needs assessment or other method (37) - Identifying stakeholders and opportunities to build internal and external partnerships (37) - Creating common goals to common outcomes, objectives, memorandum of understanding, agreement, and operating norms (37) - Developing risk and benefit analysis of the partnership (37) - Considering inequalities of power, and build organizational structures aligned with both strategy and external context (37) | - Sense of ownership of research or output (37) - Commitment to partnership - Formal training and development and the acquisition of team members’ knowledge (37) - Support from facilitators, champions, boundary spanners, or advisory boards (37) - Clear and agreed upon goals, roles, and expectations (37) | - Lack of stakeholder engagement, differing needs and priorities (37) - Unclear goals, roles and expectations (37) - Lack of funding or infrastructure of partnership, and issues of power (37) | - Developed a clear understanding of the expectations of different partners (37) - Early engagement of research users can increase accountability for research implementation at later stages (37) |
| Shared mental models | - Identifying stakeholders and opportunities to build internal and external partnerships (42) - Establishing communication methods, and conduct training and learning activities (42) | - Sense of ownership of the research and output (42) - Commitment to the partnership, formal training and development and the acquisition of team members’ knowledge and skills (42) - Positive attitude towards listening, learning, adapting and training (42) - Time for meetings for information sharing by using all-day conferences or other methods (42) - Supportive policy framework or network structure/ties for researchers and research users to create knowledge and implement research results(42) | - Lack of time for learning and training (42) - Develop relationships, build trust, and sustain intervention (42) - Performance feedback and rewards awarded to an individual when they should be awarded to a group (42) - Unclear goals, roles, and expectations (42). |  |
